# Supplementary material for: Dissociating the Effects of Light at Night from Circadian Misalignment in a Neurodevelopmental Disorder Mouse Model Using Ultradian Light–Dark Cycles
Source: Clocks Sleep. 2025 Sep 15;7(3):48. doi: 10.3390/clockssleep7030048 (PMC12452560; doi:10.3390/clockssleep7030048)
Supplement: Supplementary file 1 [file clockssleep-07-00048-s001.zip › clockssleep-3777085-supplementary.pdf]

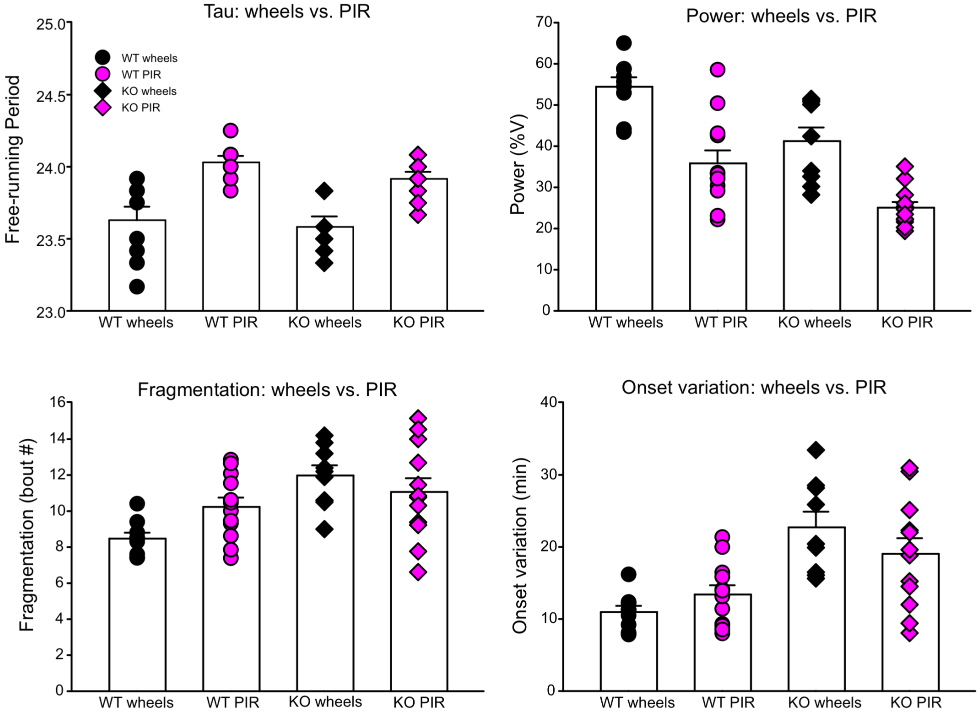

Supplementary Figure S1: Free-running period as well as other circadian parameters were measured with either running wheels or passive infrared (PIR) sensors in WT and *Cntnap2* KO mice held in constant darkness for two weeks. WT mice exhibited a significantly longer circadian period when assessed by PIR as compared to wheel-running. A similar effect of measurement method was observed in the KO mice. The same recording chambers were used to measure activity with both wheels and PIRs. These findings highlight the influence of the method used for measurement of circadian period. At least 10-days of recordings were averaged, and data were analyzed by two-way ANOVA with genotype and measurement method (wheels vs PIR) as variables. Bar graphs show the means ± SD; each dot represents an individual animal.

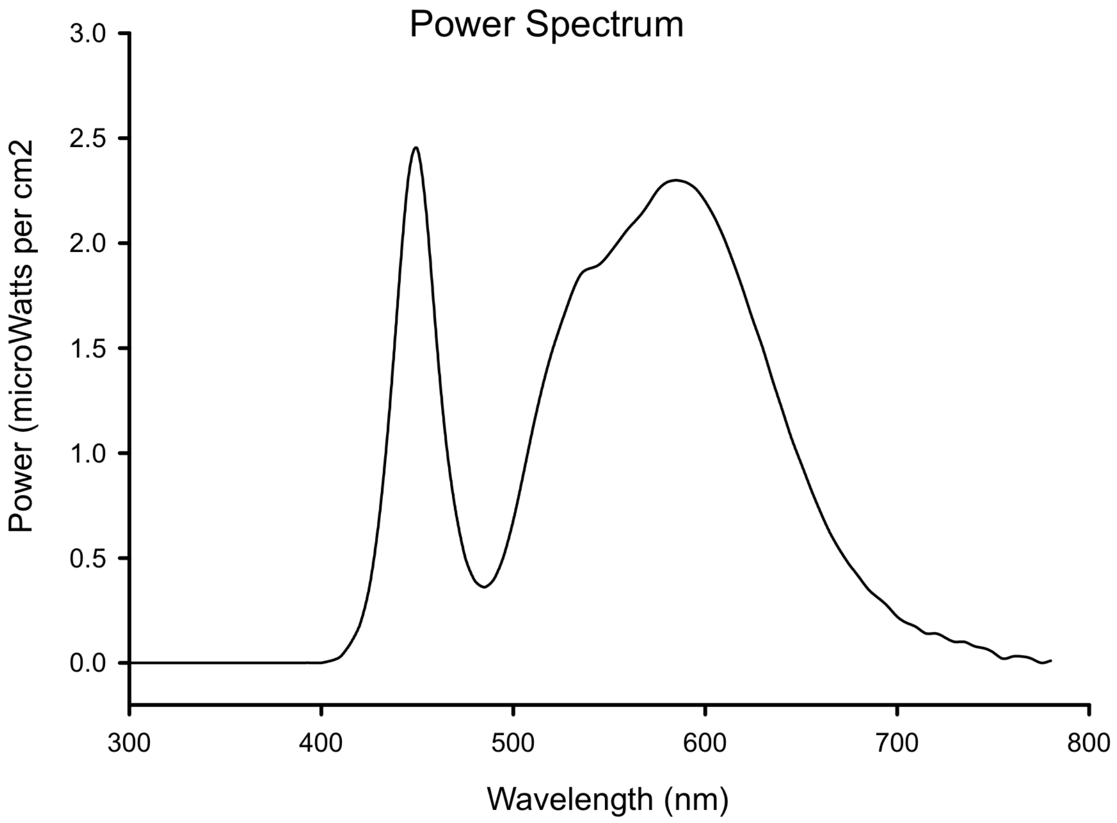

Supplementary Figure S2: The illuminance of the LED lighting was 250 lx as measured from the floor of the animal holding chamber. The irradiance was 75 microW/cm2 with a peak at 450nm and a Melanopic to Photopic (M/P) ratio of 0.57. The M/P ratios were calculated using the rodent circadian lighting toolbox courtesy of Dr. S. Peirson (Sleep and Circadian Neuroscience Institute, Oxford, UK).
